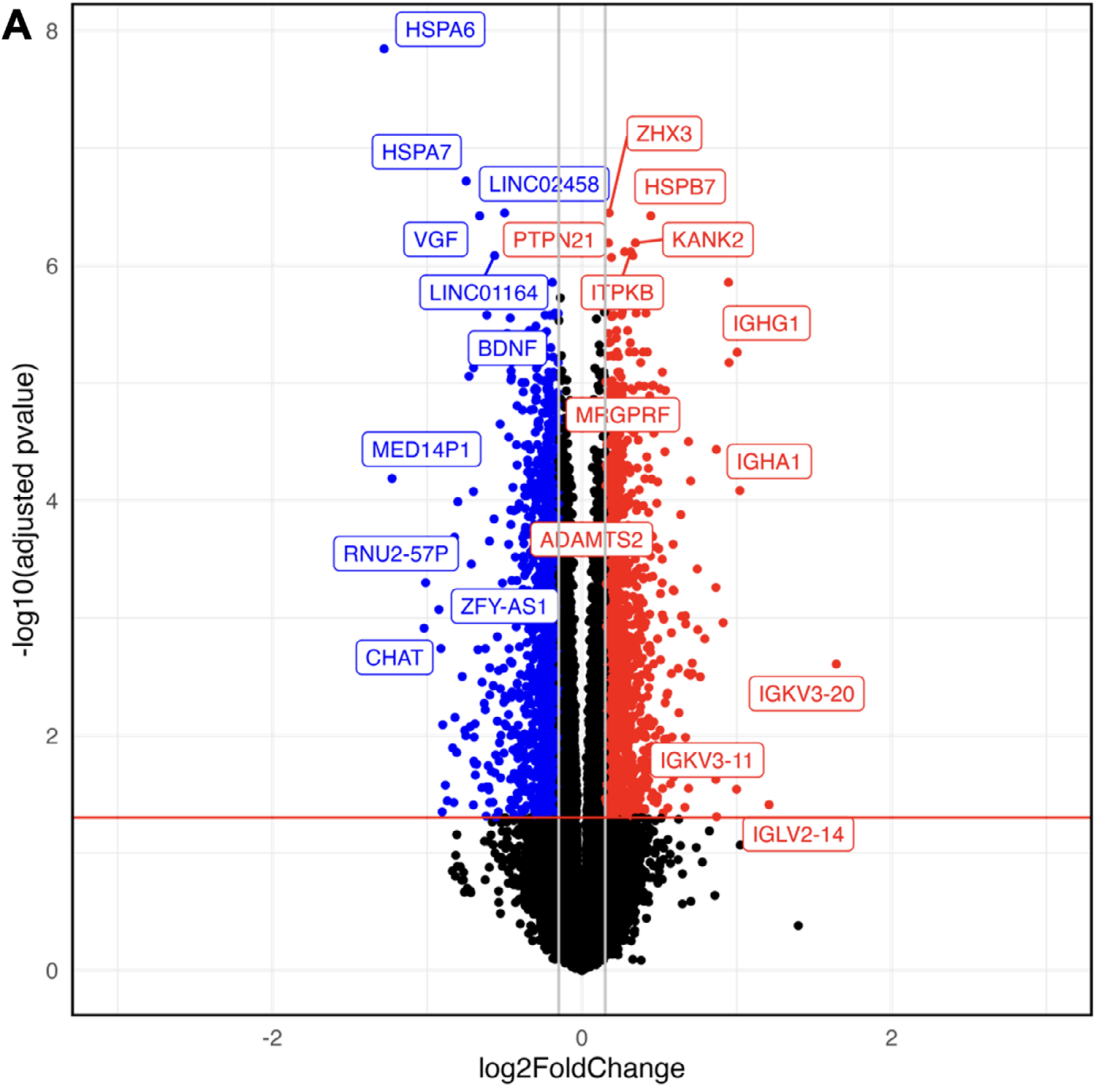# A multiethnic transcriptome for Alzheimer Disease identifies cross‐ancestry and ancestry‐specific expression profiles

**DOI:** 10.1002/alz.092011

**Published:** 2025-01-03

**Authors:** Zikun Yang, Basilio Cieza, Dolly Reyes‐Dumeyer, Annie J. Lee, Brittany N Dugger, Lee‐Way Jin, Melissa E. Murray, Dennis W. Dickson, Margaret A Pericak‐Vance, Jeffery M. Vance, Tatiana M. Foroud, Andrew F Teich, Richard Mayeux, Giuseppe Tosto

**Affiliations:** ^1^ Columbia University, New York, NY USA; ^2^ The Taub Institute for Research on Alzheimer’s Disease and the Aging Brain, Vagelos College of Physicians & Surgeons, Columbia University, New York, NY USA; ^3^ Taub Institute for Research on Alzheimer’s Disease and The Aging Brain, Columbia University Medical Center, New York, NY USA; ^4^ University of California, Davis, Sacramento, CA USA; ^5^ University of California Davis Medical Center, Davis, CA USA; ^6^ Mayo Clinic, Jacksonville, FL USA; ^7^ Department of Neuroscience, Mayo Clinic, Jacksonville, FL USA; ^8^ 1501 NW 10th Avenue, Miami, FL USA; ^9^ John P. Hussman Institute for Human Genomics, Miami, FL USA; ^10^ Department of Neurology, Indiana University School of Medicine, Indianapolis, IN USA; ^11^ Department of Pathology and Cell Biology, New York, NY USA; ^12^ Taub Institute for Research on Alzheimer’s Disease and the Aging Brain, Columbia University, New York, NY USA; ^13^ Taub Institute for Research on Alzheimer’s Disease and the Aging Brain, Vagelos College of Physicians and Surgeons, Columbia University, New York, NY USA

## Abstract

**Background:**

Alzheimer’s Disease (AD) presents complex molecular heterogeneity, influenced by a variety of factors including heterogeneous phenotypic, genetic, and neuropathologic presentations. Regulation of gene expression mechanisms is a primary interest of investigations aiming to uncover the underlying disease mechanisms and progression.

**Method:**

We generated bulk RNA‐sequencing in prefrontal cortex from 565 AD brain samples (non‐Hispanic Whites, n = 399; Hispanics, n = 113; African American, n = 12) across six U.S. brain banks, and conducted differential gene expression and enrichment analyses. We sought to identify cross‐ancestry and ancestry‐specific differentially expressed genes (DEG) and pathways across Braak stages, adjusting for sex, age at death, and RNA quality metrics. We validated our findings using the Religious Orders Study/Memory and Aging Project study (ROS/MAP, n = 1,095). Lastly, we validated top DEG using publically‐available human single‐nucleus RNA sequencing (snRNAseq) data.

**Result:**

AD‐known genes *VGF* (LFC = ‐0.661, p_adj_ = 3.78) and *ADAMTS2* (p_adj_ = 1.21) were consistently differentially expressed across statistical models, ethnic groups, and replicated in ROS/MAP (**Figure 1**). Genes from the heat shock protein (*HSP*) family, e.g. *HSPB7* (p_adj_ = 3.78), were the top DEG, also replicated in ROS/MAP. Ethnic‐stratified analyses prioritized *TNFSF14* and *SPOCD1* as top DEG in Hispanic samples. Gene set enrichment analysis highlighted several significantly pathways, including “*TYROBP* causal network in microglia” (WP3945; p_adj_ = 1.68) and “Alzheimer Disease” (WP5124; p_adj_ = 4.24). snRNAseq validated several DEG, including *VGF* downregulated in neurons (p_adj_ = 1.1).

**Conclusion:**

To our knowledge, this is the largest diverse transcriptome study for AD in post‐mortem tissue. We identified perturbated genes and pathways resulting in cross‐ethnic and ethnic‐specific findings, ultimately highlighting the importance of diversity in AD investigations.